# Two decades of coastal pressure: Assessing the effectiveness of priority conservation areas in Brazil’s Atlantic Forest

**DOI:** 10.1007/s13280-026-02403-0

**Published:** 2026-04-19

**Authors:** Agatha Simeão, Wendy Nunes Garcia, José Antonio Domingues Teixeira-Junior, David Valença Dantas, Eduardo Gentil, Marcelo Henrique Schmitz

**Affiliations:** 1 Laboratory of Geomatics and Marine Systems, Marine Management, Ecology, and Technology Group, State University of Santa Catarina, Laguna, Santa Catarina Brazil; 2 Laboratory of Marine Ecology, Marine Management, Ecology, and Technology Group, State University of Santa Catarina, Laguna, Santa Catarina Brazil; 3 Postgraduate Program in Territorial Planning and Socioenvironmental Development, State University of Santa Catarina, Florianópolis, Santa Catarina Brazil; 4 Postgraduate Program in Coastal and Lagoons Systems, State University of Santa Catarina, Laguna, Santa Catarina Brazil

**Keywords:** Biodiversity governance, Conservation planning, Land-use change, Policy effectiveness, Priority conservation areas

## Abstract

Systematic conservation planning is widely used to guide biodiversity policy, yet evidence that spatial priority setting translates into effective land-use outcomes remains limited. In Brazil, a national initiative established priority areas for conservation, sustainable use, and benefit sharing in 2004 to orient conservation investments. Two decades later, their influence on land-use trajectories remains uncertain. We assessed land-use and land-cover dynamics from 2004 to 2024 within high, very high, and extremely high priority areas in coastal municipalities of the Atlantic Forest biome. Using MapBiomas data, we quantified net and gross changes across five land-use classes and compared patterns among priority levels. Urban expansion occurred across all categories, while extremely high priority areas experienced net losses of natural vegetation and the highest land-cover turnover. These findings show that prioritization alone has limited capacity to constrain land-use pressures in dynamic coastal landscapes and requires integration with enforceable governance frameworks and territorial planning.

## Introduction

Biodiversity loss represents a critical global crisis, driven primarily by habitat destruction, fragmentation, and land-use change associated with agricultural expansion, urbanization, and infrastructure development (Pimm et al. 2014; IPBES 2019). These pressures have accelerated species extinctions and ecosystem degradation at unprecedented rates, undermining the provision of essential ecosystem services, such as carbon storage, water regulation, and climate regulation (Maxwell et al. 2016). Tropical forests, which harbor a substantial proportion of terrestrial biodiversity, have been particularly affected, with large-scale deforestation compromising both biodiversity conservation and human well-being (Foley et al. 2005).

The Atlantic Forest biome in Brazil, globally recognized as a biodiversity hotspot (Mittermeier et al. 2008), exemplifies these challenges. Currently, less than 12% of its original forest cover remains, largely due to long-term land conversion and rapid urban expansion, especially along the coast (Ribeiro et al. 2009; Rezende et al. 2018). Although international conservation agendas emphasize the role of spatial planning and protected areas in halting habitat loss, substantial gaps persist between conservation prioritization and effective implementation on the ground (Joppa and Pfaff 2011). In this context, continuous monitoring of land-use and land-cover (LULC) dynamics within priority conservation areas is essential to assess policy effectiveness and support adaptive strategies aimed at reversing biodiversity loss.

Focusing specifically on coastal municipalities allows for a more precise assessment of land-use change dynamics in Brazil’s most densely settled and economically dynamic regions, where long-term littoralization processes drive sustained pressure on conservation priorities (Ribeiro et al. 2009; Rezende et al. 2018). In these consolidated landscapes, urban expansion, rather than agricultural frontier advance, constitutes the dominant driver of land transformation, intensifying habitat loss and landscape fragmentation (Foley et al. 2005; Joppa and Pfaff 2011). Concentrating the analysis on coastal areas therefore provides a coherent framework for evaluation conservation challenges associated with established urbanization processes and persistent development pressures within priority conservation areas.

Systematic conservation planning (SCP) has emerged as a central approach to addressing such challenges, offering a structured, transparent, and replicable framework for identifying areas of high conservation value (Margules and Pressey 2000). By integrating ecological, spatial, and socioeconomic data, SCP seeks to reconcile biodiversity objectives with competing land-use demands (Kukkala and Moilanen 2013). Despite methodological advances and widespread adoption however, the long-term effectiveness of designated conservation priority areas remains uncertain, particularly in regions exposed to intense development pressures. Many prioritization initiatives yield technically robust maps and strategic plans but encounter significant obstacles during implementation, often due to limited political support, insufficient funding, or weak institutional capacity (Knight et al. 2008; Visconti et al. 2019).

Evaluating land-use trajectories within priority areas is therefore critical to determining whether spatial prioritization efforts have translated into tangible conservation outcomes or whether policy implementation remains the primary bottleneck (Foley et al. 2005; Knight et al. 2008). In this regard, Brazil occupies a prominent position in global conservation governance, hosting some of the world’s most biologically diverse ecosystems and historically advancing progressive environmental legislation and spatial planning instruments (Mittermeier et al. 2008; Scarano 2017). Nevertheless, the effectiveness of these policies ultimately depends on their translation into enforceable land-use regulation and long-term institutional commitment (Soares-Filho et al. 2010; Azevedo et al. 2017).

A major federal initiative launched in 2004 sought to strengthen biodiversity conservation through the identification of priority areas for the conservation, sustainable use, and benefit sharing of biodiversity (MMA 2004). Grounded in systematic conservation planning principles, this national process engaged researchers, policymakers, and civil society actors through extensive workshops and geospatial analyses to identify areas of high biological importance and vulnerability across all biomes. The resulting maps and technical documents were designed to guide conservation investments and support the creation or expansion of protected areas, addressing critical gaps in Brazil’s conservation network (Rodrigues et al. 2004; Joly et al. 2014).

Despite the scientific rigor and participatory nature of this prioritization effort, implementation outcomes have been uneven. Political, institutional, and economic constraints have frequently limited the translation of priority designations into concrete conservation actions, such as protected area establishment or effective land-use regulation (Castello and Macedo 2016; Fonseca and Venticinque 2018). Moreover, systematic long-term assessments of whether priority areas have maintained their ecological integrity remain scarce, hindering robust evaluations of this landmark policy instrument (Joly et al. 2014).

Against this background, a critical question remains unresolved: Have conservation priority areas influenced land-use trajectories in highly dynamic and urbanized coastal landscapes? Addressing this question is essential for understanding whether spatial prioritization has functioned as an effective governance tool or primarily as a strategic planning instrument with limited regulatory reach.

To address this gap, this study assesses the effectiveness of conservation prioritization within the Atlantic Forest biome, focusing on priority areas located in coastal municipalities. We analyze land-use and land-cover (LULC) changes between 2004 and 2024 to evaluate whether high, very high, and extremely high priority areas have experienced differential trajectories of natural vegetation retention and land-use conversion. Specifically, we examine: (1) How LULC dynamics have evolved across priority levels over two decades, and (2) whether areas identified as having higher conservation priority exhibit greater landscape stability under sustained urban pressure.

## Materials and methods

### Characterization of the study area

**Fig. 1:**
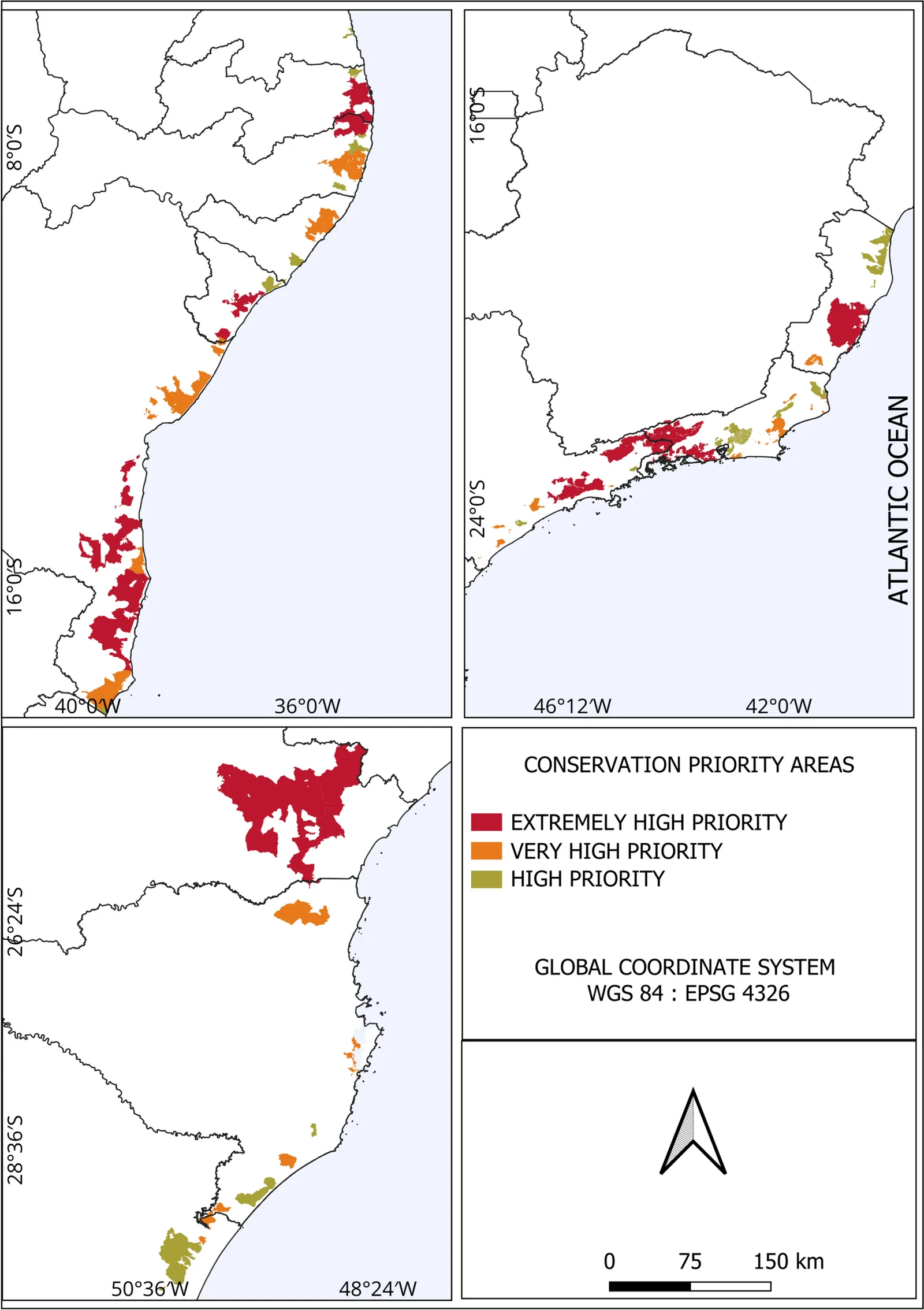
Spatial distribution of conservation priority areas within coastal municipalities of the Atlantic Forest biome, Brazil

This study was conducted within the Atlantic Forest biome and includes all priority conservation areas (PCAs) located within Brazilian coastal municipalities, as defined by the Brazilian Institute of Geography and Statistics (IBGE). The study area extends across the Northeast, Southeast, and South regions, encompassing more than 5000 km of coastline (Fig. 1). These municipalities are characterized by high ecological relevance combined with intense anthropogenic pressures, making them particularly suitable for assessing the effectiveness of conservation prioritization in highly dynamic coastal landscapes.

The priority areas for conservation (PCAs) analyzed in this study were those designated by Ordinance No. 5092/2004 of the Brazilian Ministry of the Environment. All three priority levels defined in the ordinance were included—high, very high, and extremely high—corresponding to areas of critical biological importance and high vulnerability, as established through scientific and technical assessment criteria.

### Data analysis and processing

The analysis of land-use and land-cover (LULC) changes was based on data from the MapBiomas Project, Collection 9 (MapBiomas 2025), which provides a standardized and harmonized annual time series of land-cover maps for the entire Brazilian territory at a spatial resolution of 30 m. To evaluate long-term land-use trajectories associated with the implementation of conservation priorities, we selected the years 2004 and 2024, corresponding, respectively, to the year in which the national priority ordinance was enacted and the most recent year available in the time series compatible with our analytical framework. This temporal window was chosen to enable a policy-oriented assessment of medium-term land-use outcomes rather than short-term annual variability within coastal PCAs.

For intertemporal comparison, the 38 original MapBiomas land-cover classes were reclassified into five broader categories: natural vegetation, urban area, agribusiness, water resources, and exposed soil. This aggregation was adopted to emphasize dominant land-use processes relevant to conservation and territorial planning, reduce classification noise, and ensure analytical consistency between time periods. The reclassification was applied uniformly to both years, preserving methodological coherence and avoiding distortions in the interpretation of net and gross land-cover changes.

All geospatial data processing and spatial analyses were performed using QGIS version 3.40.5. Quantitative analyses of land-cover change, including the estimation of net and gross transitions among classes, were conducted in RStudio version 4.3.

## Results

**Fig. 2:**
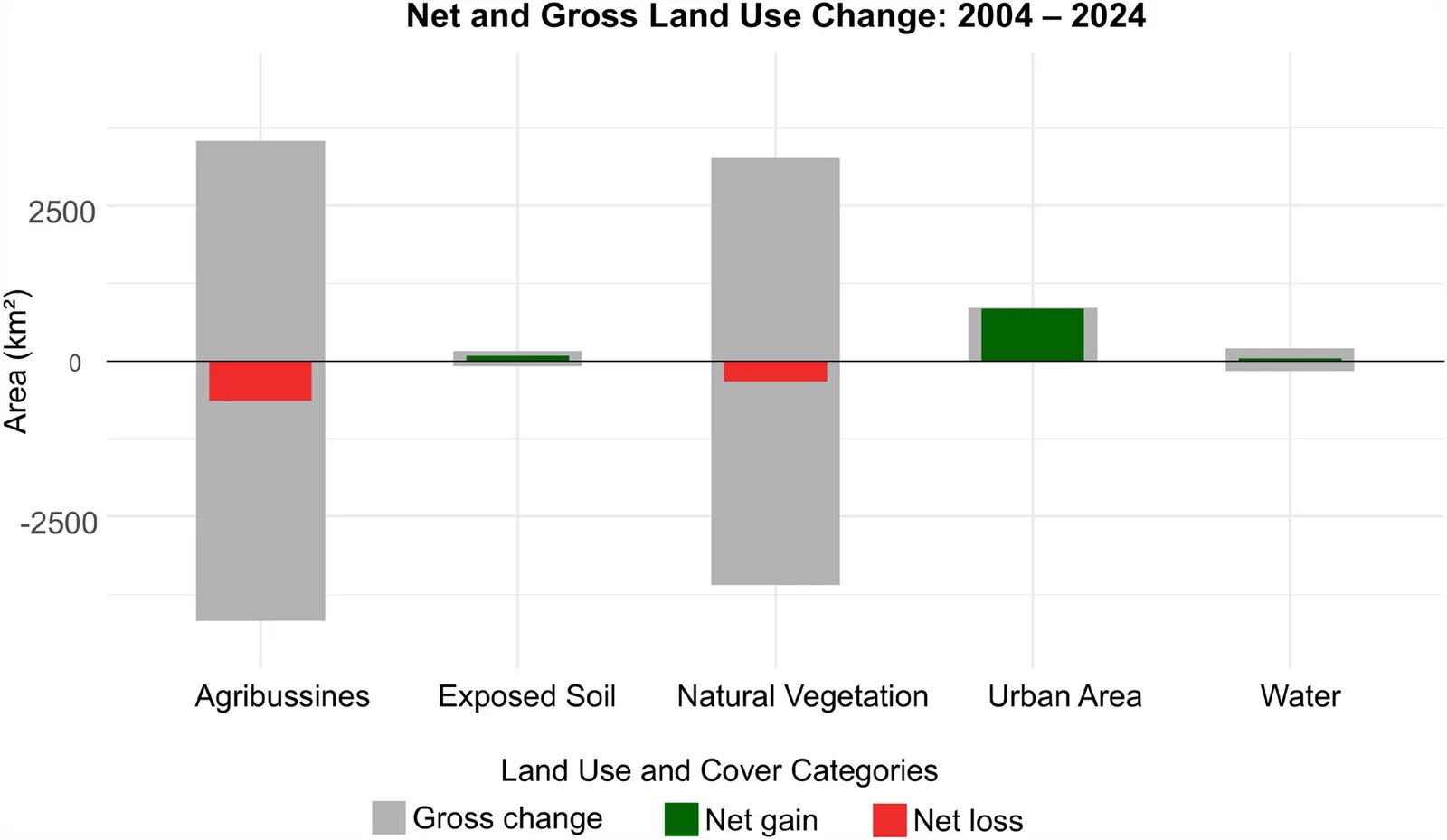
Area net gains (green), losses (red), and gross change (gray) of the land-use categories for priority conservation areas (PCAs), comparing 2004 and 2024

Cumulative land-use and land-cover transitions between 2004 and 2024 within priority conservation areas of the coastal Atlantic Forest reveal marked contrasts among land-use categories (Fig. 2). Agribusiness and natural vegetation exhibited the highest levels of gross change over the study period, indicating intense land-use turnover. Despite localized gains, both categories experienced net area losses, reflecting that conversions outpaced regeneration or reversion processes, ultimately resulting in an overall decline in natural vegetation within priority areas.

Urban area showed a consistent and positive net change, emerging as the most expansive land-use category during the period analyzed. Although its gross change was comparatively lower than that observed for agribusiness and natural vegetation, the net increase indicates a sustained and directional conversion of natural and agricultural lands into urban surfaces. In contrast, exposed soil and water resources displayed minimal net change, suggesting relative stability in their spatial extent over the two decades examined.

**Fig. 3:**
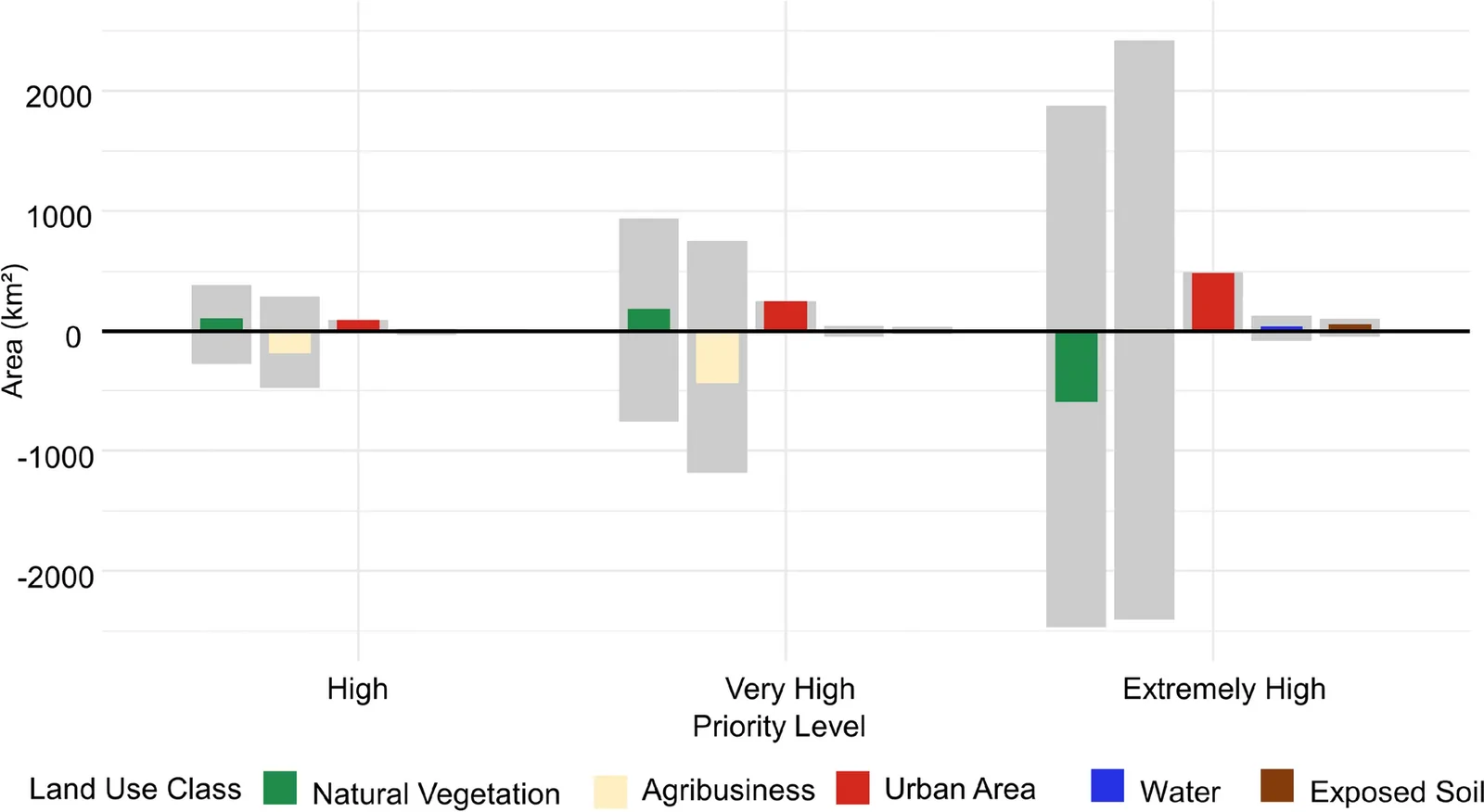
Net gains (green), losses (red), and gross change (gray) of the land-use categories for priority conservation areas (PCAs), comparing 2004 and 2024 among the three priority levels

A more detailed assessment by conservation priority level reveals distinct land-use trajectories among categories (Fig. 3). Urban area expanded consistently across all priority levels, exhibiting net gains regardless of conservation status. In contrast, the response of natural vegetation varied according to priority level. While high and very high priority areas showed net gains in natural vegetation, extremely high priority areas experienced net losses despite substantial gross change. This pattern indicates elevated land-use turnover and instability in the most critical conservation zones.

Agribusiness displayed a declining net trend in high and very high priority areas, whereas in extremely high priority areas it maintained a near-neutral net balance, even though it exhibited one of the highest levels of gross change. This suggests that, within these areas, agricultural land use was highly dynamic, with gains and losses largely offsetting one another over time. Overall, the largest gross land-cover changes were concentrated in extremely high priority areas, reflecting both their greater spatial extent and the intensity of competing land-use pressures operating within these zones.

## Discussion

Two decades after the establishment of Brazil’s Priority Areas for Biodiversity Conservation, our results provide an empirical assessment of the extent to which this national planning instrument has influenced land-use trajectories. Between 2004 and 2024, the designation of priority areas, when applied in isolation, proved insufficient to ensure the long-term maintenance of native vegetation, particularly in coastal regions subject to sustained urban pressure. Urban expansion occurred across all priority categories, while extremely high priority areas, identified as the most critical for biodiversity conservation, experienced net losses of natural vegetation alongside the highest levels of gross land-cover change. These patterns are consistent with the broader recognition that land-use change remains a dominant driver of biodiversity loss and frequently overrides conservation intentions when not counterbalanced by effective policy instruments, as highlighted by Pierce et al. (2005) in their analysis of global land-use dynamics and ecological consequences.

Although the priority-area framework is conceptually aligned with international conservation agendas, including the Convention on Biological Diversity and Sustainable Development Goal 15, our findings indicate that progress toward these global targets has remained limited. Rands et al. (2010) emphasized that ambitious biodiversity commitments require not only strategic planning but also mechanisms capable of translating priorities into concrete action. Similarly, Jenkins et al. (2015) demonstrated the spatial expansion of conservation targets alone does not guarantee effectiveness when governance structures and enforcement capacity are weak, a pattern that closely reflects the dynamics observed within Brazilian coastal priority areas.

It is therefore important to reiterate that priority areas were never intended to function as protected areas. Rather, they were conceived as strategic, science-based instruments designed to guide public policy, direct conservation investments, and support decisions regarding the creation or expansion of protected areas. This distinction is central to interpreting the outcomes observed in this study. Adams et al. (2019) argued that systematic conservation planning plays a critical role in informing policy and resource allocation, but its effectiveness ultimately depends on the degree to which priorities are embedded within binding regulatory and institutional frameworks. In the Brazilian context, our results suggest that, in the absence of such frameworks, priority areas largely fulfilled their informational role but had limited influence on territorial outcomes.

The disconnect between planning and implementation identified here aligns with the well-documented research–implementation gap in conservation science. Knight et al. (2008) showed that conservation planning frequently produces technically robust spatial priorities that fail to influence land-use decisions when political, legal, and economic constraints are not adequately addressed. Likewise, Nori et al. (2013) demonstrated that priority-setting exercises have limited capacity to prevent habitat loss unless supported by enforceable land-use regulations and sustained institutional commitment. Our findings provide empirical support for these arguments, indicating that priority designation alone did not alter dominant land-use trajectories in highly dynamic coastal landscapes.

Despite these limitations, the national priority-setting process represents a significant governance milestone. It was national in scope, multisectoral, participatory, and grounded in systematic conservation planning principles, conferring technical legitimacy and broad institutional recognition (MMA 2004). However, translating this legitimacy into effective conservation outcomes requires governance arrangements capable of addressing the complex socioecological contexts in which priority areas are embedded. Schmitz et al. (2023) underscored that conservation effectiveness increasingly depends on integrating environmental objectives with broader territorial and development policies, particularly in regions undergoing rapid socioeconomic change. This challenge is especially pronounced along the Atlantic Forest coast, where conservation priorities intersect with urban expansion, tourism, infrastructure development, and diverse local economic interests (Ribeiro et al. 2009; Rezende et al. 2018).

Land-use dynamics within priority areas further illustrate this complexity. The differentiated responses observed across priority levels, combined with high rates of gross land-cover change even in areas showing localized vegetation gains, indicate land-use instability and rotational dynamics rather than sustained conservation trajectories. Similar patterns have been reported in coastal and forested landscapes where competing land uses and fragmented governance structures constrain long-term conservation effectiveness (Lira et al. 2012; Moraes et al. 2017; Lima and Ranieri 2018). These findings reinforce the argument advanced by Watson et al. (2018) that conservation in human-dominated landscapes requires governance systems capable of managing trade-offs among multiple actors and scales.

In Brazil, protected areas continue to play a central role in conservation policy, particularly in coastal and marine contexts. As documented by Ribeiro et al. (2020), coastal and marine protected areas have expanded substantially, contributing to biodiversity conservation and territorial management. National assessments have also highlighted the positive economic contributions of protected areas to the Brazilian economy (Medeiros and Young 2011). Nevertheless, our results suggest that, despite the recent expansion of the National System of Protected Areas, these efforts remain insufficient to achieve conservation targets across all biomes within established timelines. Achieving national and international biodiversity commitments will therefore require moving beyond the identification of priority areas toward governance arrangements and policy instruments capable of operating within the complex and contested realities of socioecological landscapes.

## Conclusion

Two decades after Brazil’s national conservation priority-setting initiative, our findings demonstrate that spatial prioritization, when not coupled with binding policy instruments, has limited capacity to influence land-use trajectories in highly dynamic coastal landscapes. High and extremely high priority areas continued to experience urban expansion and, in the most critical zones, net losses of natural vegetation. These patterns indicate that conservation intent has been consistently outweighed by prevailing development pressures. Importantly, these outcomes do not reflect a conceptual limitation of systematic conservation planning, but rather constraints associated with its implementation within fragmented governance and regulatory contexts.

Priority areas were designed to inform decision-making, guide conservation investments, and support the creation or expansion of protected areas, rather than to function as direct land-use restrictions. However, the absence of enforceable regulatory mechanisms, sustained institutional capacity, and effective integration with territorial planning has restricted their ability to shape long-term conservation outcomes. Our results highlight the need to embed conservation priorities within coherent governance frameworks that align environmental objectives with urban planning, infrastructure development, and local economic dynamics.

From a policy perspective, achieving national and global biodiversity targets will require moving beyond the identification of priority areas toward governance instruments capable of managing trade-offs in human-dominated landscapes. Strengthening regulatory coherence, enhancing cross-sectoral coordination, and ensuring long-term monitoring are essential steps to transform conservation priorities from strategic planning tools into effective mechanisms for biodiversity governance, particularly in rapidly transforming coastal regions.

## Data Availability

Data will be made available on reasonable request.
